# Analysis of Acousto-Optic Phenomenon in SAW Acoustofluidic Chip and Its Application in Light Refocusing

**DOI:** 10.3390/mi14050943

**Published:** 2023-04-26

**Authors:** Xianming Qin, Xuan Chen, Qiqi Yang, Lei Yang, Yan Liu, Chuanyu Zhang, Xueyong Wei, Weidong Wang

**Affiliations:** 1 School of Mechano-Electronic Engineering, Xidian University, Xi’an 710071, China; qinxianming@xidian.edu.cn (X.Q.);; 2 CityU-Xidian Joint Laboratory of Micro/Nano-Manufacturing, Xi’an 710071, China; 3 State Key Laboratory for Manufacturing Systems Engineering, Xi’an Jiaotong University, Xi’an 710049, Chinachuanyu.zhang@xjtu.edu.cn (C.Z.)

**Keywords:** acoustofluidics, acousto-optic effect, SAW chip

## Abstract

This paper describes and analyzes a common acousto-optic phenomenon in surface acoustic wave (SAW) microfluidic chips and accomplishes some imaging experiments based on these analyses. This phenomenon in acoustofluidic chips includes the appearance of bright and dark stripes and image distortion. This article analyzes the three-dimensional acoustic pressure field and refractive index field distribution induced by focused acoustic fields and completes an analysis of the light path in an uneven refractive index medium. Based on the analysis of microfluidic devices, a SAW device based on a solid medium is further proposed. This MEMS SAW device can refocus the light beam and adjust the sharpness of the micrograph. The focal length can be controlled by changing the voltage. Moreover, the chip is also proven to be capable of forming a refractive index field in scattering media, such as tissue phantom and pig subcutaneous fat layer. This chip has the potential to be used as a planar microscale optical component that is easy to integrate and further optimize and provides a new concept about tunable imaging devices that can be attached directly to the skin or tissue.

## 1. Introduction

Surface acoustic wave (SAW) devices based on piezoelectric materials and interdigital electrodes (IDTs) have a long history and many applications, such as radars [1], filters [2], gratings [3], and acousto-optical modulators [4]. In recent years, SAW devices have been further applied to the field of acoustofluidics [5,6]. Based on microelectromechanical systems (MEMS) technology, SAW acoustofluidic devices can realize precise operation [7] on droplets, particles, and cells [8,9,10,11,12,13,14] in a noninvasive, label-free, and contactless manner, making it a powerful and advanced tool in fields such as biology [15], chemistry [16], and forensic analysis [17].

The technology of SAW acoustofluidic devices is gradually maturing, but there are still many phenomena that have not been explained. For example, in some acoustofluidic experiments, strange light and shade phenomena appear in the place where ultrasonic waves act [18]. Unlike acousto-optic modulators, these shades are caused by light perpendicular to the chip plane instead of parallel [19]. SAW devices are generally considered able to construct a stable standing field on the piezoelectric substrate plane [20], thus changing the refractive index of the waveguide to achieve an acousto-optic effect. However, in acoustofluidic devices, Rayleigh waves generated by SAW transducers leak into the fluid in the form of inhomogeneous waves. On the one hand, with complex acoustic pressure distribution in three dimensions, analysis of the acousto-optic effect in SAW acoustofluidic devices is more complicated, but on the other hand, it has the potential to realize different modulation performances based on SAW. However, instead of tunable lenses [21,22], SAW optical devices are mostly used as deflectors [23] or frequency shifters [24]. Unlike SAW devices, bulk wave (BW) acousto-optical devices can refocus or modulate light sources into different patterns or even clarify an image [25,26,27,28].

In this paper, we explain the acousto-optic phenomenon in SAW acoustofluidic devices with a light beam refraction simulation. Based on these analyses, a new kind of MEMS SAW acousto-optic device was developed. Modified from a focused surface acoustic wave (FSAW) microfluidic chip, this device can modulate the refractive index of the bonded medium and refocus the light beam perpendicular to its SAW plane to clarify the image. With its performance verified with scattering media, such as tissue phantom and porcine epithelium, this device has the potential to be integrated into microscale optical equipment and provides a new concept for in vivo acousto-optic imaging applications involving skin and other biological tissues.

## 2. Phenomenon and Analysis

### 2.1. Analysis of the Focused Acoustic Field

**Figure 1:**
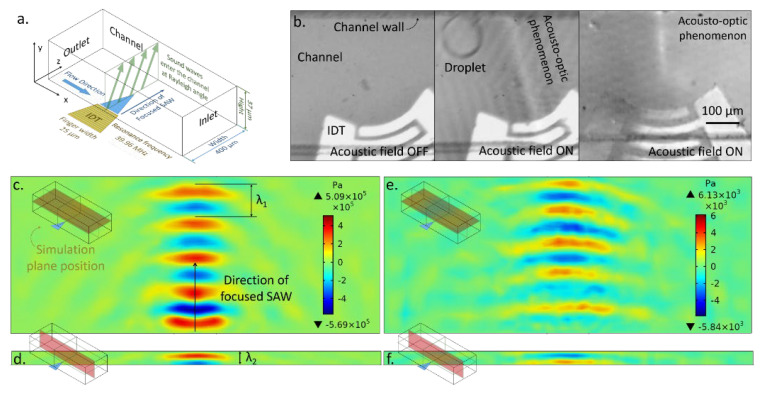
Analysis of the focused acoustic field: (**a**) Schematic diagram of the FSAW microfluidic device; (**b**) acousto-optic phenomena in SAW microfluidic devices; (**c**,**d**) acoustic pressure field distribution; (**e**,**f**) time-averaged acoustic pressure field distribution. The pink rectangle in the schematic diagram on the left of (**c**–**f**), respectively, refers to the plane where the simulation results are located in the channel.

In SAW acoustofluidic devices, the position of the acousto-optic phenomenon is consistent with the position of the acoustic field at resonance [20,29,30]. Bright stripes are located in the center of the acoustic field along the propagation direction of the SAW beam, while darker stripes are distributed around it (Figure 1b–d). To analyze the acousto-optic phenomenon, we first simulated the three-dimensional distribution of the acoustic field in the fluid.

In the COMSOL simulation of the acoustofluidics experiment, the substrate is 128-degree YX-cut lithium niobate. As shown in Table 1, the size of the substrate is 1500 μm × 1200 μm × 200 μm (length × width × thickness). The substrate thickness is set to 300 μm. The sound velocity on the surface of lithium niobate is 3996 m/s. The IDTs are modeled as perfect conductors using boundary conditions. Assuming the IDTs are very thin compared with the piezoelectric substrate, the effect of their mass and stiffness on the dynamics of the device is not accounted for. The model is solved at a target frequency of 39.96 MHz. The applied AC voltage is 35 V. The finger width and interspace of the IDTs are 25 μm. The acoustic window is 45°. The number of electrodes is set as 4 for computational efficiency. The simulation cannot calculate the coupling of the acoustic waves generated by all 50 electrodes, so it can only roughly show the distribution of the acoustic pressure field. The channel is 1000 μm × 37 μm × 475 μm (length × height × width). The channel is set to pure water at room temperature.

The substrate is set as a piezoelectric material with a mechanical damping. The loss factor defined in the frequency domain at the target frequency is converted to the Rayleigh stiffness damping parameter. The bottom surface and all the surfaces on the sides of the substrate are assigned as the low-reflecting boundary condition. The microchannels are polydimethylsiloxane (PDMS) grooves processed with photolithography technology. The channel roof and channel walls are set with an acoustic impedance of 1070 kg/m^3^ × 1030 m/s Pa·s/m, which reflects the PDMS boundary. In the simulation, the multiphysics interface “fluid–structure interaction” and “acoustic–structure boundary” are used to model the interaction between elastic waves in solids and pressure waves in fluids.

After a SAW enters the channel, it constructs a three-dimensional acoustic field in the liquid [31]. The wavelength λ1 of the SAW on the piezoelectric substrate is 100 μm, influenced by the IDT pattern, signal frequency, and properties of the substrate. When the sound wave enters the fluid medium inside the channel, the change in sound speed in the medium will lead to a change in wavelength. In the fluid, sound waves propagate in the form of an inhomogeneous wave with wavelength λ2, 37 μm. As shown in Figure 1, in the X-direction, i.e., the flow direction, the width of the acoustic pressure field obeys the size of the acoustic window of focusing IDT. In the Y-direction, i.e., the height direction, the high- and low-pressure regions are alternately distributed, with λ2 as the period. In the Z-direction, that is, in the SAW propagation direction and the direction of the angular bisector of the IDT, the high- and low-pressure regions of the acoustic pressure field are alternately distributed with λ1 as the period. There is a time scale difference between the flow in the channel, MHz acoustic waves, and beam propagation. For flow analysis, the acoustic field is time-averaged, while for light analysis, the acoustic field is static. Therefore, the transient simulation analysis can reflect the acoustic field through which the light passes, while the time-average acoustic field simulation can reflect the actual acoustic field distribution acting on the microfluidic targets. In Figure 1e,f, the time-averaged result is the distribution of the average acoustic pressure in ten periods. In the microfluidics experiments in Figure 1, the fluid is deionized water. The flow rate is regulated by a pressure pump (LSP01-2A, LongerPump, CN).

### 2.2. The Analysis of the Phenomenon through Simulation

The density of the fluid medium is modulated by the pressure waves generated from the SAW. The medium in the high-pressure region is compressed, leading to an increase in density, whereas, in the negative-pressure region, the opposite is true. The refractive index will change with the density. Therefore, the fluctuation of the acoustic pressure field will change the local refractive index of the medium as follows [32]:

(1) $$n^{2}=1+\sum_{i=1}^{3}\frac{(\alpha_{i}P^{2}+\beta_{i}P+\delta_{i})\lambda_{L}^{2}}{\lambda_{L}^{2}-(A_{i}P^{2}+B_{i}P+\Delta_{i})}$$

where $\alpha_{i}$, $\beta_{i}$, and $\delta_{i}$ and the corresponding Greek capital letters A_*i*_, B_*i*_, and Δ_*i*_ are the coefficients of the polynomials describing the Sellmeier dispersion coefficients considered pressure-dependent. n is the refractive index, and *λ_L_* is the wavelength of the light. The study of Maysamreza Chamanzar [27] simplifies the relationship between the refractive index and pressure. With the fixed wavelength and temperature, the refractive index distribution can be calculated from the simulated pressure curve by using the following linear relationship:

(2) $$n_{local}=n_{0}+kP_{local}$$

where $n_{local}$ is the local refractive index, $n_{0}$ is the refractive index of the medium at room temperature (25 °C) in the visible optical wavelength range, $P_{local}$ is the local pressure, and k is an empirical coefficient. For water at room temperature, k is 1.402 × 10^−5^ bar^−1^ [33].

Based on the pressure distribution and the simplified relationship between the refractive index and pressure, the distribution of the refractive index can be calculated with the Runge Kutta method in MATLAB [34]. The calculation is performed on the X–Y planes, perpendicular to the propagation direction of SAW (Z-direction). The position of the plane is determined by the distance $d_{p}$ from the plane to the channel wall.

Firstly, we model the refractive index field in MATLAB according to Equation (2). Since the relationship is linear (Equation (2)), the refractive index distribution simulated in MATLAB is very similar to the acoustic pressure field in the COMSOL simulation. To balance the accuracy and computation time, the simulation plane is divided into a 300 (X-direction) × 100 (Y-direction) grid, and all data points on the grid are initialized. The actual size of a single grid is about 3.3 × 0.37 μm. The Barron interpolation method is used to convert discrete index data points into continuous index fields on the grid. The refractive index gradient values of all data points in the X and Y directions are calculated, and a uniformly grided refractive index gradient field data is modeled.

Secondly, the light path is simulated in MATLAB. Light rays are set as incident perpendicular to the bottom of the channel. The step size of the Runge Kutta method is partially determined by the mesh size of the refractive index field. In addition, the step size varies according to the change in the refractive index. When the refractive index changes greatly, the step size will become smaller to avoid distortion. When the change in the refractive index exceeds 10^−7^, 10^−6^, 10^−5^, and 10^−4^, the step size will be reduced to 0.5, 0.2, 0.1, and 0.05 of the original step size, respectively. The number of rays is 321. There is an incident ray at x = 0 μm (that is, the location of the center line of the IDT), and 160 rays on its left and right side, respectively. The incident points of these 321 rays are uniformly distributed on the *X*-axis (bottom of the channel) and are within the range of x = (−500, 500) μm. For the light in a certain unit of the grid, the direction change is calculated based on the local refractive index gradient, and the optical path length is calculated based on the variable step size, so the displacement vector and position data are obtained. The Runge Kutta method is used to continuously calculate the position of the light rays until they reach the grid boundary.

**Figure 2:**
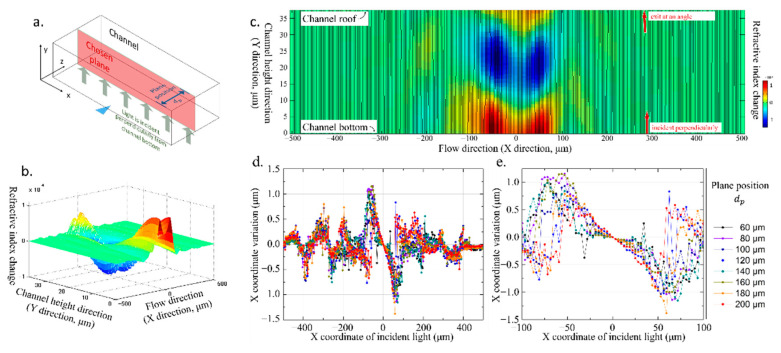
Simulation of refractive index distribution and optical path: (**a**) the X–Y plane selected for simulation, perpendicular to the bottom of the channel and the propagation direction of SAW; (**b**) the refractive index distribution on the selected plane; (**c**) the simulation result of 321 light paths on the selected plane; (**d**) the displacement of the light path on the *X*-axis; (**e**) a partial close-up of (**d**), the 200 μm wide central area around x = 0.

Finally, the optical paths on multiple X–Y planes within the channel are simulated. The light enters vertically from the bottom of the channel, propagates in the liquid medium with a variable refractive index, and exits at the channel roof (Figure 2). To better understand the light path in the whole channel area, several vertical planes with different $d_{p}$ are selected, and every light beam on each plane is simulated. The change in the X-coordinate from the incident position to the exit position of each light path is calculated (Figure 2d,e). It can be seen that in the two neighborhoods around the middle (x = 0), there is a peak on the left and a trough on the right, while the part near the center quickly approaches zero. This shows that in this variable refractive index field, the light on the left and right sides tends to approach the middle. The original uniformly distributed light converges to the center under the influence of the variable refractive index. In addition, in the area far away from the acoustic line, that is, in the range of about (−500, −100) μm and (100, 500) μm, the light path also has some small variation. In these areas, the pressure in the fluid is not constant, but the amplitude of the acoustic pressure is low, so the change in the refractive index is small. Although the X-coordinate variation in light is no more than 2 μm in the simulation, it is only the result of the change in light inside the microfluidic channel. The position change of the X-coordinate of light shows the change in the light’s direction. The outgoing light from the top of the channel will go through a long light path till it forms a micro photo in the camera, which makes the original small change become an obvious acousto-optic phenomenon.

## 3. Application Experiments

**Figure 3:**
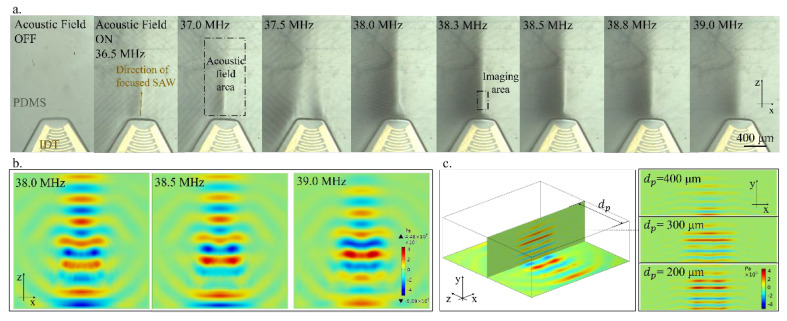
The SAW chip with PDMS as solid media: (**a**) acousto-optic phenomenon patterns under different frequencies; (**b**) the acoustic pressure distribution in X–Z plane under different frequencies; (**c**) the acoustic pressure field in the X–Y planes with different $d_{p}$.

To utilize and verify the analysis demonstrated above, we propose a SAW chip that can adjust focus. Manufactured with the MEMS process, this chip retains the design of focused IDT but does not retain any microchannels; instead, a 5 mm thick PDMS layer is used as the medium to conduct sound waves and light waves. In this way, the robustness of the device can be increased, and more importantly, pressure pumps or other microfluidics equipment are no longer needed to control the liquid. Under the action of a focused acoustic field, the acousto-optic phenomenon produces different patterns under different frequencies (Figure 3). From 38.0 MHz to 39.0 MHz, the change in vibration modes on the piezoelectric substrate influences the distribution of the refractive index inside PDMS. The voltage applied is 20 V.

Both the finger width and interspace of the IDT are 25 μm. The designed resonant frequency of the IDT transducer on the piezoelectric substrate is 39.96 MHz, but after bonding with PDMS, the actual resonant frequency will change to around 38.3 MHz. The acoustic window is 45°, and the geometric focal length is 200 μm. The amount of electrode finger pairs will affect the acoustic energy density and resonant frequency bandwidth. In order to form resonance, the number of finger pairs should not be more than 100. On this chip, each transducer has 50 pairs of electrodes. The chip manufacturing method and experimental equipment are the same as previously published articles [30].

For SAW chips with solid media in Figure 3, its acoustic pressure field is simulated in COMSOL (Figure 3b,c). The distribution of the acoustic pressure field in fluids (Figure 1) and elastic polymers (Figure 3) exhibits high similarity. In the X–Z plane, the focused acoustic wave beam (Figure 3b) determines the acoustic field area (Figure 3a), while in the X–Y plane, the propagation of acoustic waves generates a pressure field (Figure 3c) that modulates the refractive index field. Due to the situation that one of the most critical parameters, the Gladstone–Dale coefficient of PDMS, has not been reported before, the specific refractive index change in PDMS under the acousto-optic effect can hardly be calculated by theoretical simulation. However, according to the experimental results, it can be observed that the SAW chip with PDMS as the medium can effectively excite acousto-optic phenomena. As shown in Figure 3, the acousto-optic phenomenon exhibits different patterns (Figure 3a) under the acoustic fields at different frequencies (Figure 3b). In Figure 3a, the wavelength range of the light source is 400–760 nm, and the direction of the light is perpendicular to the X–Y plane. In the COMSOL simulation of Figure 3b,c, the modeling method is similar to the simulation in Figure 1, but the fluid medium with a 600 × 200 × 600 μm (length × height × width) PDMS cube is replaced, and the acoustic impedance boundary condition is replaced with a low reflection boundary.

**Figure 4:**
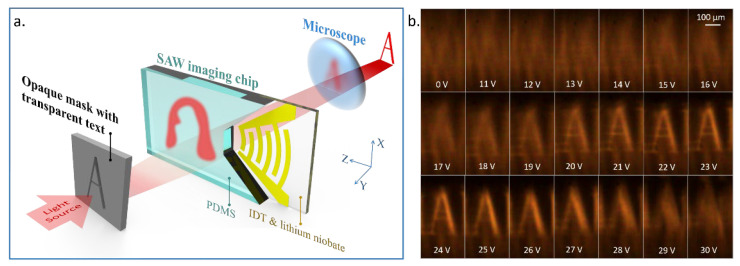
The method of testing the new SAW chip and its performance. (**a**) Schematic diagram of device structure and imaging method. (**b**) Images modulated by acoustic fields with different voltages.

We used a very simple and straightforward method to demonstrate the MEMS SAW device’s refocus ability. The chip is put on an opaque mask with transparent characters, through which the light source can form luminous characters (Figure 4). A microscope with a focal length of 45 mm is used to observe the characters’ image and the modulation effect. First, the mask is put on the focal point, and the image is clear. Then, in order to imitate a blurred image, the mask is deliberately moved a certain distance away from the objective lens along the Y-direction to cause the image of the luminous characters to be out of focus. Finally, the acoustic field is turned on, and the voltage is adjusted until the light ray refocuses. In Figure 4b, the distance between the mask and the focal point is 1200 μm. As the voltage increases, the letter A in the image first gradually becomes recognizable and then blurs again, which demonstrates the process of equivalent focal length changing with an increase in acoustic field intensity. The size of the imaging area is subject to the region of the acoustic field. In the X-direction, the width of the imaging area is determined by the acoustic aperture. In the Z-direction, the length of the imaging area depends on the acoustic waves’ attenuation length.

**Figure 5:**
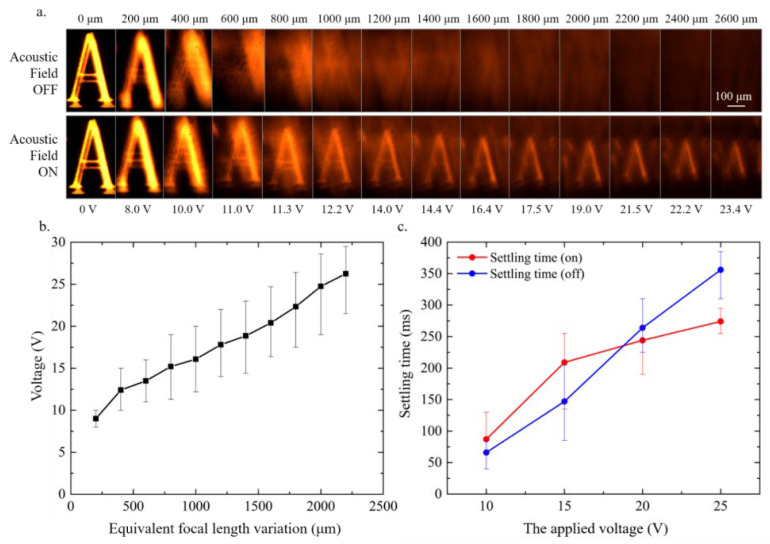
Relationship between voltage and device performance: (**a**) for every two images in the column, the data above are the distance between the mask and the focal point, and the data below are the voltage applied on the transducer; (**b**) the average result of the relationship between voltage and equivalent focal length variation; (**c**) the relationship between applied voltage and settling time. The settling time (on) and the settling time (off) refer to the time from power turned on or off to the stabilization of the image, respectively.

The equivalent focal length can be adjusted by changing the voltage to control the intensity of the acoustic field (Figure 5). We used chips with the same design in the experiments to test their refocus effect. The relationship between the equivalent focal length variation and the voltage is approximately linear (Figure 5b). A linear relation exists between the applied voltage of the transducer responsible for exciting the ultrasonic resonance and the induced acoustic pressure amplitude [35], which results in a linear relationship between the voltage and the local refractive index, according to Equation (2). Each data point is the average of four measurements. The device has a settling time when the acoustic field is turned on. This settling time is not decided by the sound velocity. The acoustic field needs a certain amount of time to accumulate before the amplitude of the acoustic pressure field reaches the maximum and stability. Similarly, when the power is turned off, it also takes a certain period of time for the acoustic field to gradually decline before it disappears due to the acoustic waves’ attenuation. With an increase in voltage, no matter whether the power is on or off, the settling time will be prolonged (Figure 5c). There is a linear relationship between acoustic energy density and the square of voltage [35], which results in a direct proportional relationship between the settling time and voltage. Each data point is the average of five tested settling times.

**Figure 6:**
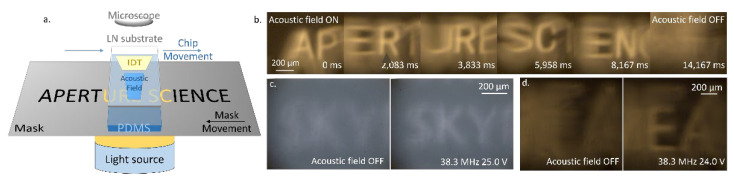
Experiments with dynamic image and scattering medium: (**a**) schematic diagram of chip position and movement; (**b**) experimental results of dynamic image modulation; (**c**) experiments with tissue phantom as scattering medium; (**d)** experiments with pig subcutaneous fat layer as scattering medium.

Because the MEMS SAW chip is based on the planar structure of a lithium niobate (LN) wafer, it can be put in different positions or on different media to modulate the image. Firstly, we tested the chip’s performance for dynamic images. By moving the chip and mask in the opposite direction, the light in different regions is refocused (Figure 6a,b). The voltage applied to the device is 24.7 V, and the distance between the mask and the focal point is 1600 μm. Since the PDMS waveguide bonded to the transducer is flexible, the chip has a good conformal ability, which makes it adaptable on uneven surfaces. The video of the dynamic image modulation is in the Supplementary Materials.

Secondly, we tested the chip’s performance on different materials. Scattering media, such as human skin and tissue, can disperse the light beams passing through, making the captured image blurred. Ultrasonic waves undergo minimal attenuation (about 0.3~0.6 dB cm^−1^ MHz^−1^) when propagating through biological tissue [36,37], which makes it possible for the SAW chip to refocus the light beam inside skin or tissue by pasting the transducer directly on them. In the experiment, we used titanium dioxide mixed with PDMS as tissue phantom, which can simulate the human epithelium’s optical properties and has tunable reduced scattering and absorption coefficients at visible and near-infrared wavelengths [36,37]. Although the scattering coefficient and absorption coefficient of tissue phantom (TiO_2_/PDMS) are different from transparent medium (pure PDMS), their similarity in acoustic characteristics makes it possible for SAW to modulate the refractive index distribution and sharpen the image (Figure 6c,d). For the chip with tissue phantom, the concentration of titanium dioxide is 3 ‰, and the reduced scattering coefficient and absorption coefficient are about 5 cm^−1^ and 0.03 cm^−1^, respectively. The thickness of the tissue phantom is about 2 mm. As a solid medium and a replacement for PDMS, tissue phantom is directly bonded to the piezoelectric substrate using oxygen plasma bombardment.

In addition to tissue phantom, some biological tissues were used for similar experiments (Figure 6d). The subcutaneous fat layer of pigskin (Oxygen Crown, CN) is cut into about a 1 mm thick layer and placed between the device and the mask as a scattering medium. Usually, the characters can hardly be recognized under the pig fat layer. After the acoustic field is turned on, some characters can be vaguely distinguished. However, it should be noted that the fat layer is only pressed on the chip instead of bonded. If the chip is tightly bonded to the adipose layer or other similar tissue so that sound waves can construct a better index field inside, the imaging quality will be better.

## 4. Discussion

The principle of the acousto-optic effect in acousto-optical modulators and SAW microfluidic devices is the same, but the relative position of light and acoustic field is different, resulting in different acousto-optic phenomena. In SAW acousto-optic modulators, such as tunable SAW optical switches, light propagates along the surface plane of the piezoelectric substrate. For SAW microfluidic devices, the beam is perpendicular to the SAW plane and travels in the three-dimensional acoustic pressure field, resulting in different performances of light modulation. Based on the analysis of the three-dimensional refractive index field and light path, a new kind of SAW imaging device was developed, and its performance was tested through a series of experiments.

This MEMS SAW device can extend the focal length of the microscope within the range of 0~2000 μm. Although within the range of 2000~2600 μm, it can still achieve light refocusing, but the image is difficult to recognize due to its small size. Although the device cannot directly change the resolution of an image, the sharpness of the image can be adjusted by controlling the voltage. The imaging speed of this device is influenced by voltage, and the image settling time varies from 50 to 300 ms in a voltage range of 10~30 V. The size of the imaging area of this device is controlled by an acoustic aperture width in the X-direction and an acoustic field in the Z-direction, which is approximately 200 μm (X-direction) × 600 μm (Z-direction).

Compared with a tunable lens [25], this surface acoustic wave device aims at an in vivo imaging device. Although it has a zooming effect, its purpose is not to replace the lens but to change the refractive index inside the observed object and turn the target into an equivalent convex lens.

Compared with a cylindrical acousto-optic relay lens [26], the acousto-optic interaction distance of this SAW chip is short. The acousto-optic interaction distance of a cylindrical acoustic relay lens can reach tens of millimeters [27], while the interaction distance in our SAW chip depends on the attenuation of the acoustic field, that is, the height of the acoustic field in the Y-direction. Its short acousto-optic interaction distance causes the maximum acousto-optic modulation effect of our chip to be weaker than cylindrical ones as a sacrifice to balance its size and performance. However, the cylindrical acousto-optic relay lens is based on hollow cylindrical acoustic transducer arrays, so the target sample needs to be wrapped in a hollow cylinder to achieve ultrasonic sculpting of virtual optical waveguides, which limits the size and type of the sample to be observed [28]. For example, a cylindrical ultrasonic transducer cannot be used for samples larger than the cylinder. In addition, the planar structure of this device allows it to be attached directly to the target sample’s surface, allowing it to be further optimized to work as a planar microscale optical component that is easy to integrate.

## 5. Conclusions

The acousto-optic phenomena in acoustic microfluidic devices are quite different from those in acousto-optic modulators. In this paper, we analyzed the refractive index field that an acoustic wave constructs in a medium and explained the phenomenon. Based on the analysis, a new kind of SAW chip was developed that can refocus the light to make an originally blurred image clear. Through a series of experiments, the relationship between the voltage and equivalent focal length variation was established. Experiments with the dynamic image and tissue phantom showed that this device has the potential to be used in in vivo imaging, such as observing fluorescently labeled tissues that are blocked by scattering media.

## Figures and Tables

**Table 1:** COMSOL simulation parameters.

Parameter Name	Parameter Value
Designed frequency	39.96 MHz
Applied voltage	25 V
Finger width and interspace of the IDT	25 μm
Size of piezoelectric substrate	1500 μm × 1200 μm × 200 μm
Size of channel	1000 μm × 37 μm × 475 μm
Maximum calculation step time	1.25 × 10^−9^ s

## Data Availability

Not applicable.

## Supplementary Materials

The following supporting information can be downloaded at: https://www.mdpi.com/article/10.3390/mi14050943/s1, Video S1.
